# Sonoclot® predicts operation time and blood loss after cardiopulmonary bypass in children

**DOI:** 10.1016/j.heliyon.2022.e11461

**Published:** 2022-11-09

**Authors:** Hidehisa Saito, Shin Kawana, Kazutomo Saito, Ayuko Igarashi, Mari Inokuchi, Masanori Yamauchi

**Affiliations:** a Department of Anesthesiology and Perioperative Medicine, Tohoku University Graduate School of Medicine, Sendai, Miyagi, Japan; b Department of Anesthesia, Miyagi Children's Hospital, Sendai, Miyagi, Japan; c Department of Surgical Operations, Tohoku University Hospital, Japan

**Keywords:** Sonoclot, Bleeding, Pediatric cardiac surgery, Fluid and transfusion therapy, Coagulation function, Cardiopulmonary bypass

## Abstract

**Background:**

As the circulating blood volume is relatively small in pediatric patients, blood components are quickly lost when bleeding, which make it more difficult to stop the bleeding. Particularly in pediatric cardiac surgery, loss of clotting factors associated with cardiopulmonary bypass (CPB) would likely to be prominent. As a result, bleeding is further promoted and the operation time is extended. In order to search for the relation between clotting factors and the amount of bleeding, we used a viscoelastic point of care test.

**Objectives:**

We used Sonoclot® as viscoelastic point of care test to evaluate coagulation function before CPB and before weaning from CPB in pediatric cardiac surgery.

**Design:**

Retrospective.

**Setting:**

Single-institutional.

**Participants:**

We included 55 pediatric patients (median age 13 months [IQR 5–32]) who underwent cardiac surgery under CPB from December 2015 to November 2016.

**Interventions:**

None.

**Measurements and main results:**

Sonoclot® analysis was performed after induction of anesthesia (pre-data, or baseline data) and before any heparinized saline was given, and right after modified ultrafiltration after weaning from CPB (post-data). Post-data was compared with post-CPB operation time and post-CPB blood loss by multiple regression analysis. Furthermore, effects of fresh frozen plasma (FFP) added on CPB on coagulation function and post-platelet function (describe as PF_SC_) was assessed. Activated coagulation time (describe as ACT_SC_) and clot rate (describe as CR_SC_) showed no significant change between baseline data and post-data. Post-PF_SC_ was worsened by prolonged CPB time (p < 0.05) and correlated to bleeding amount and operation time after CPB (p < 0.05). Total amount of platelet concentrate (PC) transfused was higher in patients with smaller PF_SC_ (p < 0.05). Total amount of FFP and PC transfused correlated with bleeding amount and operation time after CPB (p < 0.05). In the subgroup analysis, PF_SC_ declined in the FFP-included group, whereas there was no significant difference in coagulation function. Addition of FFP to CPB did not significantly affect CR, whereas PF_SC_ deteriorated as CPB time was prolonged (CPB time = 1/(0.0021∗PF_SC_ + 0.0055)).

**Conclusion:**

Sonoclot® is useful to evaluate coagulation function in pediatric patients who undergo CPB. Preventive administration of FFP or PC in CPB circuit could reduce bleeding after CPB.

## 1 Introduction

Coagulation function is one of the main factors to determine operation time and blood loss after cardiopulmonary bypass (CPB). The more complicated the congenital heart disease, the more sophisticated the repair that is required in pediatric cardiac surgery. Especially in pediatric cases, imperceptible but significant bleeding at sites of suture or dissection can sometimes be difficult to control [1, 2]. In addition, as the circulating blood volume is relatively small, blood components are quickly lost when bleeding, which make it more difficult to stop the bleeding. A deterioration in coagulation function after CPB would further prolong the operation time, and as a result, increase the rate of complications [3]. Therefore, evaluation of coagulation function is important to establish an appropriate replacement strategy [4].

Recently, some coagulation monitors such as TEG® and ROTEM® have been introduced, and these results has been proven by these tools which enable the measurement of coagulation function at the bedside in a timely manner [5, 6, 7, 8]. However, there are few reports which demonstrated consistent data using Sonoclot® (SC, Sienco, US) as a viscoelastic point of care test. The basic principle of measurements using the Sonoclot® is to detect changes in viscoelasticity of whole blood following coagulation. Sonoclot® needs only a 0.36-mL blood sample for measurement and provides data on the activated coagulation time, clot rate, and platelet function [9]. Using a glass bead cuvette with heparinase enables the measurement of coagulation function, even during CPB [10]. Vishwakarma et al. and Dominique et al. found that postoperative bleeding can be predicted by using Sonoclot® in pediatric cardiac surgery [11, 12]. However, we anesthesiologists would expect to predict the amount of blood loss during surgery.

In this study, we retrospectively evaluated coagulation function before CPB and just before weaning from CPB, and analyzed the relationship to the post-CPB operation time and blood loss in pediatric cardiac surgery.

## 2 Materials and methods

The ethics committee of Miyagi children's hospital approved this retrospective study. The requirement for informed consent was waived due to the study's retrospective design. Fifty-five pediatric patients who underwent cardiac surgery under CPB from December 2015 to November 2016 were involved. Patient characteristics are presented in Table 1.

**Table 1:** Patient characteristics.

	Total
Number of patients	55
Sex (male/female)	33 (60%)/22 (40%)
Age (months)	13.0 (5.0–32.0)
Height (cm)	74.1 (57.2–88.0)
Weight (kg)	8.3 (4.6–11.0)
Type of operation (ASD, VSD/CoA/DORV/EA/HLHS/PA/TGA/TOF)	22/3/6/3/3/7/5/6
CPB time (min)	147 (107–224)
Operating time after CPB (min)	83 (69–116)
Blood loss after CPB (mL)	34.0 (18.5–76.5)
Blood loss after CPB per patient weight (mL/kg)	4.0 (1.6–8.5)
Total amount of FFP transfused (mL/kg)	43.0 (10.3–83.0)
Total amount of PC transfused (mL/kg)	0.0 (0.0–72.0)

Values are median (interquartile range).

ASD: atrial septal defect, VSD: ventricular septal defect, CoA: coactation of aorta, CPB: cardiopulmonary bypass, DORV: double outlet right ventricle, EA: Ebstein's anomaly, FFP: fresh frozen plasma, HLHS: hypoplastic left heart syndrome, PA: pulmonary atresia, PC: platelet concentrate, TGA: transportation of hate great arteries, TOF: tetralogy of Fallot.

Standard monitoring for cardiac surgery was applied and peripheral arterial catheter was cannulated to measure blood pressure directly, so as to analyze blood gas and Sonoclot® data. In cases of a complicated anomaly, FFP was added to the CPB circuit beforehand by the request of cardiac surgeons. A blood transfusion including RBC, FFP, and PC following CPB was performed at the discretion of the attending anesthesiologist.

Sonoclot® analysis was performed after induction of anesthesia with glass beads cuvette (pre-data, or baseline data) before any heparinized saline was given, and right after modified ultrafiltration after weaning from CPB with heparinase-added glass beads cuvette (post-data).

**Figure 1:**
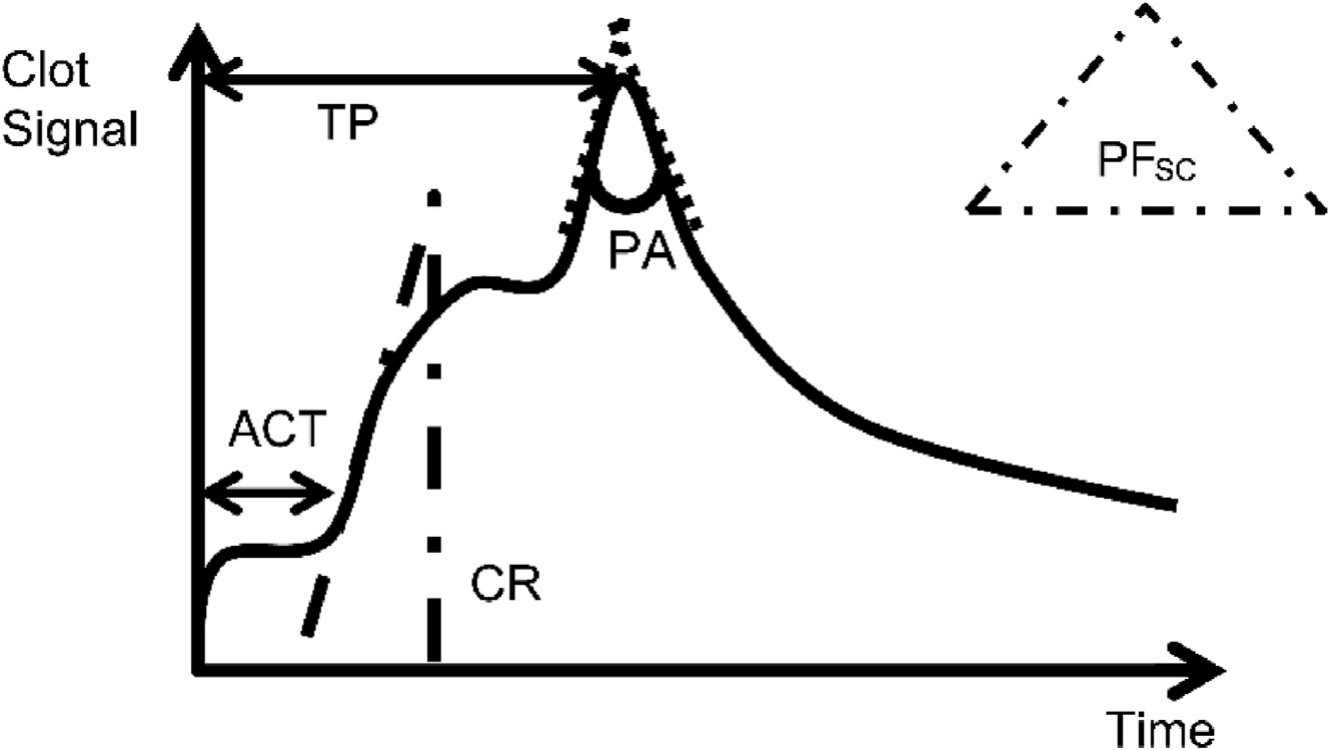
Example graph obtained by the Sonoclot® (SC). Activated clotting time (ACT) is the time until the blood coagulation starts. Clot rate (CR) shows the coagulating speed from when the coagulation starts. These two parameters represent the clotting function. Meanwhile, time-to-peak (TP), peak angle (PA), and platelet function of SC (PF_SC_) represent the platelet function. TP is the time until clot retraction when platelets agglutinate. PA is the angle of vertex, which indicates the clotting speed of TP. PF_SC_ is used to assess the platelet function comprehensively by using TP and PA.

The Sonoclot® monitor provides the following parameters: activated clotting time (ACT_SC_, which needs protamine administration when it becomes longer), clot rate (CR_SC_, which needs FFP administration when it becomes smaller), and platelet function (PF_SC_, which needs platelet administration when it becomes smaller, especially less than 1.2). More specifically, ACT_SC_ is the time to the start of the coagulation cascade activated by the glass beads in a cuvette. CR_SC_ is calculated from the slope of the Sonoclot® curve representing initial fibrin polymerization and clot development. Platelets are activated following thrombin formation in a glass bead cuvette. In the case of a normal blood sample, platelet coagulation makes a sharp secondary peak on the Sonoclot® curve due to the acute increase in the viscoelasticity of the sample, followed by an acute decrease due to the detachment of the sample from the surface of the cuvette (Figure 1). The time-to-peak (TP) represents the time to complete secondary coagulation by the platelets. The peak angle (PA) represents the cohesiveness of the clot by secondary coagulation.

PF_SC_ is a provisional parameter that is calculated from a closed algorithm using PA and TP (Figure 1). The number of PF_SC_ is decreased when PF is deteriorated, and the cut-off value is 2 according to the Sienco Co.

We compared Sonoclot® baseline data with post-data to evaluate the deterioration in coagulation function by CPB. Secondarily, we compared post-data with the post-CPB operation time and post-CPB blood loss to reveal the effects on coagulation function at the end of CPB, because most time would have been spent controlling the bleeding after CPB. Therefore, bleeding after CPB was used for this study. We defined post-CPB operation time as time from cessation of CPB to skin closure. Even if the amount of bleeding is the same, it is expected that the meaning will be completely different depending on the body surface area, so the amount of bleeding was converted to the amount per body weight. We also analyzed the relationship between post-PF_SC_ and the total amount of blood transfusion. In addition, the effects of FFP-added CPB on coagulation function and post-PF_SC_ was surveyed as a subgroup analysis.

### 2.1 Statistical analysis

Mann-Whitney's U test and Pearson's chi-square test were used to compare patient characteristics in the amount of bleeding after CPB and post-PF_SC_. Mann-Whitney's U test was used to compare baseline data and post-data of Sonoclot®. In addition, it was used to assess PF_SC_ and clotting function after CPB, and the length of CPB time in both FFP-included and FFP-not included CPB. Post-Sonoclot® data and CPB time, operation time, and amount of bleeding after CPB were compared by Logistic multiple regression analysis. JMP Pro 14 for Windows (SAS Institute, Cary, NC, USA) was used for all statistical processing and graph generation. Testing was two-tailed at a 5% significant level.

## 3 Results

**Figure 2:**
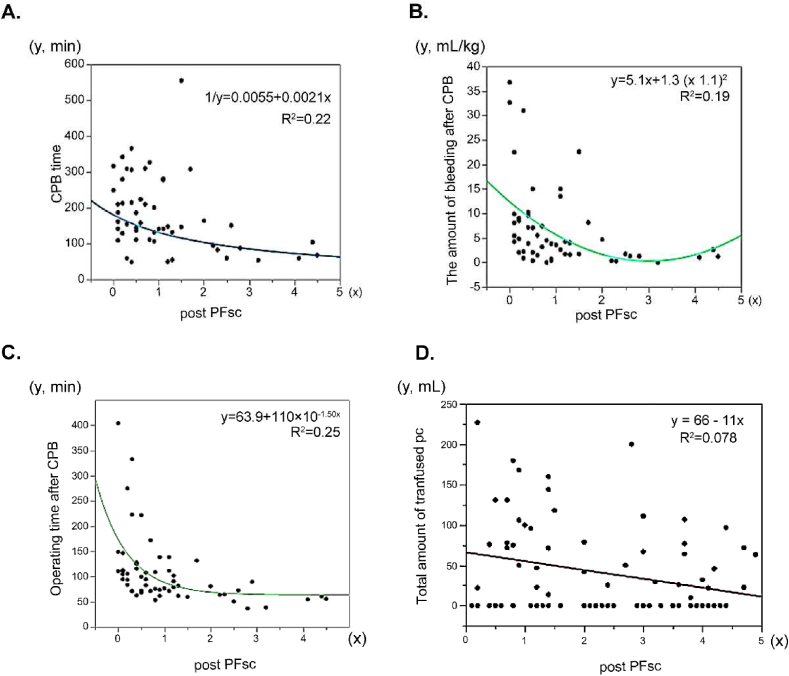
Correlations with PF_SC_ (A) Post-PF_SC_ and CPB time had a negative correlation (p < 0.05). Its regression formula was 1/y = 0.0021x + 0.0055 (R^2^ = 0.22) (B) Post-PF_SC_ and the amount of bleeding after CPB had a significant correlation (p < 0.05). Its regression formula was y = 5.1x + 1.3 (x-1.1)^2^ (R^2^ = 0.19) (C) Post-PF_SC_ and operating time after CPB had a significant correlation (p < 0.05). The regression formula was y = 63.9 + 110 × 10–1.50x (R^2^ = 0.25) (D) Post-PF_SC_ and the total amount of platelet concentrate transfusion had a significant correlation (p < 0.05). The regression formula was y = 66–11x (R^2^ = 0.078).

Table 1 shows the demographics of the patients. Median age was 13 months old, and size of patients was 55. Median operating time after CPB was 83min, and blood loss after CPB was 34mL, or that per patient weight was 4 mL/kg. Baseline data and post-data of Sonoclot® are compared in Table 2. There was no significant difference in ACT_SC_ and CR_SC_. On the contrary, platelet function, represented as PF_SC_, significantly worsened from 2.4 to 0.8 (p < 0.05, respectively). Thereafter, we analyzed PF_SC_ as a representative parameter for platelet function in Figures 1 and 2.

**Table 2:** Sonoclot® (SC) data before and after CPB.

	Baseline data	Post-data	p-value
ACT	150.5 (130.8–191.3)	161.0 (143.0–190.0)	0.20
CR	24.0 (19.0–30.3)	21.0 (14.9–28.0)	0.28
PA	63.0 (36.8–107.0)	123.0 (76.0–144.0)	<0.05
TP	621.0 (492.0–797.3)	685.0 (599.0–963.0)	<0.05
PF_SC_	2.4 (1.2–3.8)	0.8 (0.3–15.0)	<0.05

ACT: activated coagulation time, CR: clot rate, PA: peak angle, TP: time-to-peak, PF: platelet function.

In Figure 2A, post-PF_SC_ was shown to be significantly worsened by a prolonged CPB time (p < 0.05). In Figures 2B and Fig. 2C, post-PF_SC_ was significantly correlated with the amount of bleeding and the operation time after CPB (p < 0.05). The regression formula in each figure shows the correlation between them. It suggests that when the PF_SC_ became below the standard value of 2, the amount of bleeding after CPB significantly increased, and the operation time was prolonged accordingly.

According to Figure 2D, the total amount of PC transfused was significantly higher among patients whose PF_SC_ was smaller.

**Figure 3:**
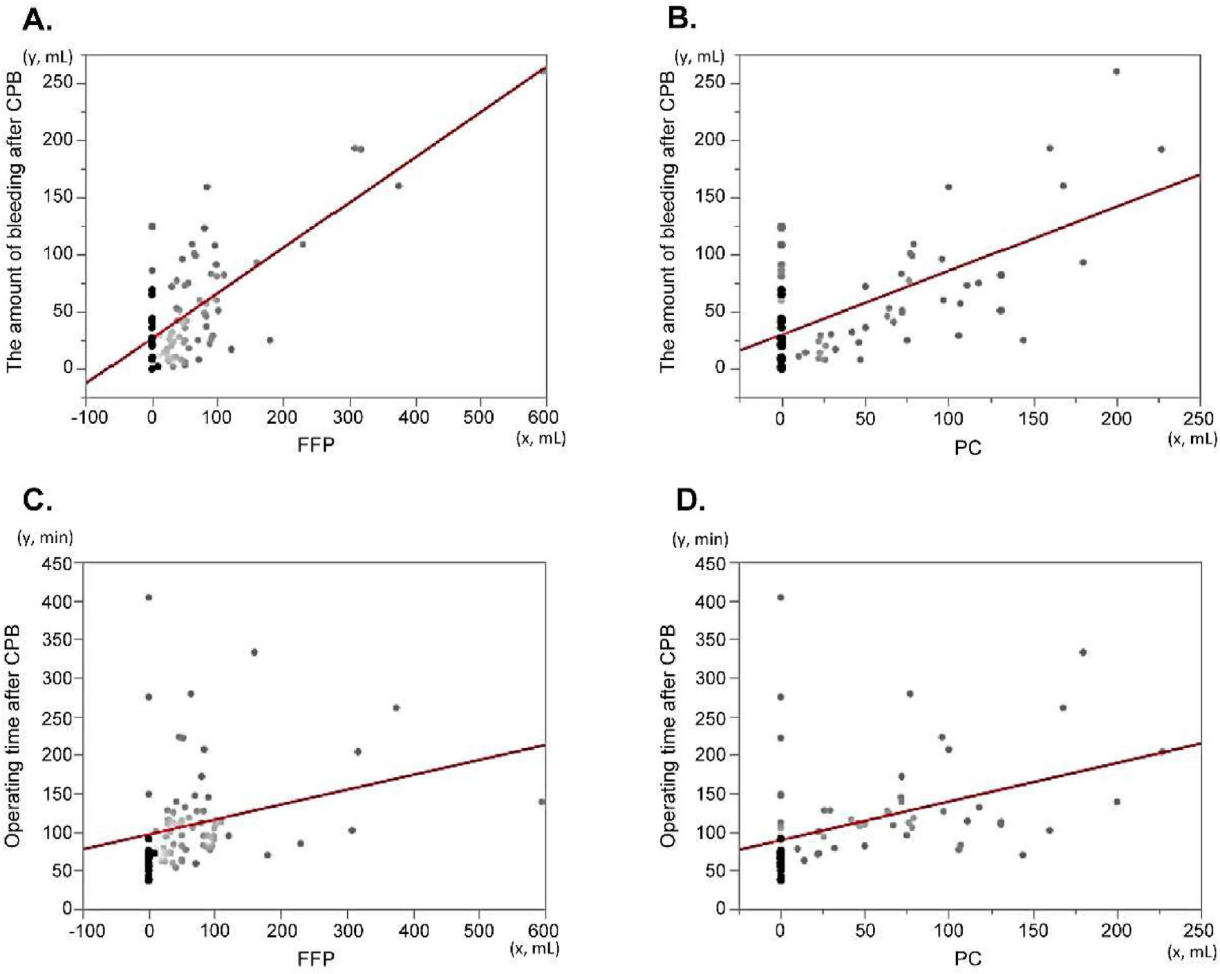
Total amount of FFP and PC transfused correlated with the amount of bleeding after CPB and operating time after CPB (p < 0.05). The regression formulae of each graph. FFP and the amount of bleeding after CPB was y = 26.5 + 0.40x (R^2^ = 0.52), PC and the amount of bleeding after CPB was y = 29.7 + 0.56x (R^2^ = 0.39), FFP and operating time after CPB was y = 96.7 + 0.19x (R^2^ = 0.072), and PC and operating time after CPB was y = 89.2 + 0.50x (R^2^ = 0.18).

In Figure 3, the total amount of FFP and PC transfused was significantly correlated to the amount of bleeding and the operation time after CPB (p < 0.05).

**Figure 4:**
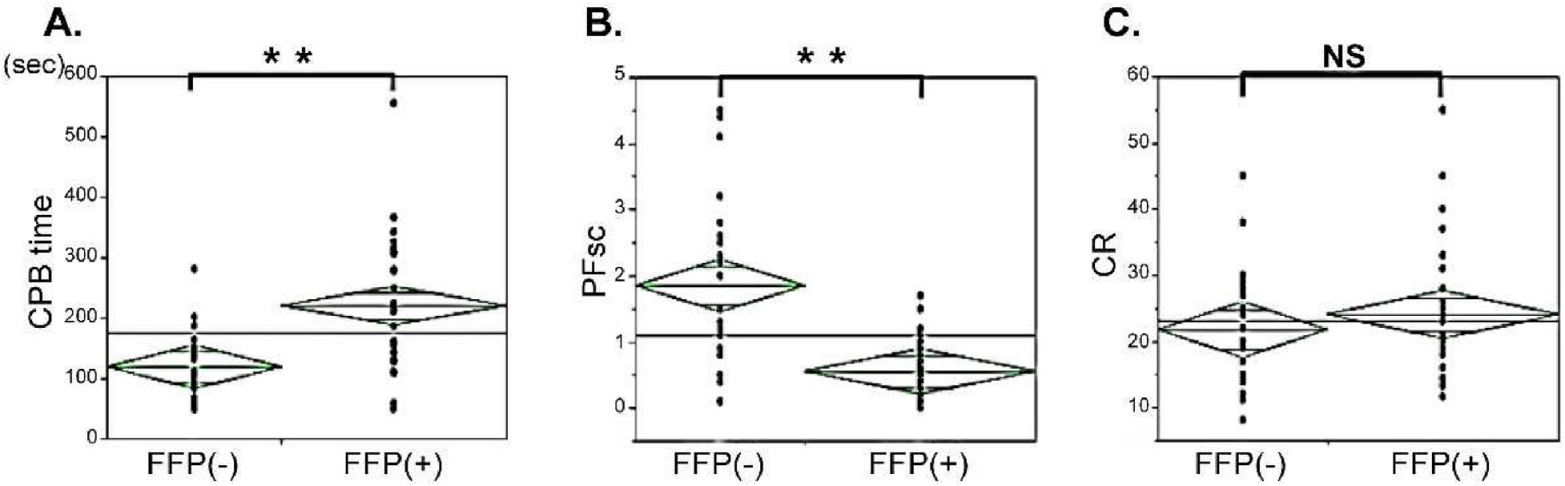
The effect of FFP inclusion in CPB on CPB time, post PF_SC_, and CR (∗∗p < 0.05).

For a closer look at the effect of FFP added to the CPB circuit, subgroup analysis was performed. Figure 4 shows that PF declined in the FFP-included group, whereas there was no significant difference in coagulation function.

## 4 Discussion

In this study, we showed that CPB did not worsen ACT_SC_ but significantly deteriorated PF_SC_ in pediatric cardiac patients regardless of FFP addition in CPB beforehand. The amount of bleeding and operation time after CPB had a significant correlation with post-PF_SC_. Anita et al. [13] and Varghese et al. [14] reported that platelet dysfunction after CPB is caused by a defect of its surface glycoproteins such as GMP-140. Tetsuya et al. reported that Sonoclot® had a significant correlation with the amount of bleeding and blood transfusion during and after adult cardiac surgery [15]. The results of this study indicate that Sonoclot® can also be useful in pediatric cardiac surgery to predict the amount of bleeding and for fluid and transfusion therapy.

Figure 2 shows that platelet dysfunction after CPB correlated with the amount of bleeding, the operating time after CPB, and the total amount of PC transfusion. Kelsey et al. [16] reported that the use of a coagulation monitor helped in the decision-making process regarding appropriate platelet transfusion in their institutions.

However, we did not measure the PC count in this study. Thus, it is not clear if PF_SC_ deterioration was due to a decreased number of platelets, or the decline in platelet function itself, or both. Hofer et al. [17] reported that platelet count, platelet component, and PF decreased by more than 30% after CPB; both of the above could be the reason. Meanwhile, Gregorio et al. [18] reported that pediatric patients are particularly prone to CPB-induced coagulopathy mainly due to hemodilution, consumption of coagulation factors, and hypothermia, and that postoperative bleeding in children undergoing cardiac surgery with CPB was linked to younger age, longer CPB duration, and a significant postoperative reduction in platelet count and function. Therefore, in any case, it is certain that replacement of preserved platelets and reduction of CPB time are important.

The response of Sonoclot® values to transfusion therapy was not evaluated. Figure 3 shows a positive correlation between the amount of bleeding, or operating time, after CPB, and the total amount of transfused FFP or PC. There was, however, a possibility that FFP or PC was infused to maintain preload, not as hemostatic agents. The amount of transfused FFP or PC and their infusion speed was dependent on anesthesiologists in charge. To establish an appropriate strategy, the endpoint of replacement of FFP and PC will be determined by Sonoclot® in the future.

According to the result of the subgroup analysis, which is shown in Figure 4, the addition of FFP to CPB did not have a significant effect on CR, while PF_SC_ deteriorated as CPB time was prolonged. If coagulating function declined as CPB time was prolonged, CR should also have declined, though this was not the result. This suggests that it might be useful to keep coagulating function constant in the FFP-added group. On the other hand, Sandrine et al. [19] reported that PC-coating of the CPB circuit made it possible to reduce early postoperative bleeding. Ten coronary artery bypass graft surgery patients and 10 mitral valve repair patients were randomized either to the PC-coated CPB or uncoated CPB groups. Although no significant difference between groups was observed in the markers of platelet activation or thrombin generation, blood loss was significantly lower in the PC coated group during the first 6 h postoperatively. Moreover, Siemens et al. [20] reported that antifibrinolytic drugs, such as aprotinin, tranexamic acid, and epsilon-aminocaproic acid, reduced 24-h blood loss and blood product transfusion. These results suggest that preventive administration of FFP or PC in the CPB circuit might be useful to reduce bleeding after CPB.

This study has some limitations. A blood count, including PC, at the time of Sonoclot® measurement was not performed. Furthermore, the response of Sonoclot® values to transfusion therapy was not evaluated.

In conclusion, the present study showed that Sonoclot® was a useful monitor to evaluate coagulation function in pediatric patients who have undergone cardiac surgery under CPB.

## 5 Limitation

Some results drawn in this review are based on retrospective study or small sample size. Studies of larger sample size and more multicenter, randomized controlled trials should be conducted to evaluate the clinical efficacy of Sonoclot®.

## Declarations

### Author contribution statement

Hidehisa Saito, Assistant Professor: Conceived and designed the experiments; Performed the experiments; Analyzed and interpreted the data; Wrote the paper.

Shin Kawana: Conceived and designed the experiments; Performed the experiments; Analyzed and interpreted the data; Contributed reagents, materials, analysis tools or data; Wrote the paper.

Kazutomo Saito, Assistant Professor; Masanori Yamauchi, Professor: Contributed reagents, materials, analysis tools or data; Wrote the paper.

Ayuko Igarashi; Mari Inokuchi: Performed the experiments; Wrote the paper.

### Funding statement

This research did not receive any specific grant from funding agencies in the public, commercial, or not-for-profit sectors.

### Data availability statement

Data will be made available on request.

### Declaration of interest's statement

The authors declare no conflict of interest.

### Additional information

No additional information is available for this paper.
